# Tetrahydroxystilbene Glucoside Suppresses NAPDH Oxidative Stress to Mitigate Apoptosis and Autophagy Induced by Cerebral Ischemia/Reperfusion Injury in Mice

**DOI:** 10.1155/2019/3913981

**Published:** 2019-07-17

**Authors:** Feng Yu, Wei Xue, Liuyi Dong, Xiangyang Hu, Dake Huang, Kai Wang

**Affiliations:** ^1^Department of Neurology, The First Affiliated Hospital of Anhui Medical University, Hefei 230022, China; ^2^Department of Neurology, Anhui Provincial Hospital, Hefei 230001, China; ^3^Department of Pharmacology, Anhui Medical University, Hefei 230032, China; ^4^Department of Pathology, Anhui Medical University, Hefei 230032, China; ^5^Synthetic Laboratory of Basic Medicine College, Anhui Medical University, Hefei 230032, China

## Abstract

Tetrahydroxystilbene glucoside (TSG) is the active ingredient extracted from the traditional Chinese medicine* Fallopia multiflora*, which has extensive pharmacological activities. The current study aimed to observe the neuroprotective mechanism of TSG in the ischemia/reperfusion (I/R) brain injury-induced apoptosis and autophagy from the point of view of oxidative stress (OS). The middle cerebral artery occlusion (MCAO) model was prepared through the suture-occluded method, and TSG was administered through tail vein injection at the time of reperfusion at the doses of 3.0, 6.0, and 12.0 mg/kg. Compared with sham group, the neurological score in I/R mice was increased (P<0.05), along with remarkably elevated cerebral infarct volume (P<0.05); while TSG administration could reduce the neurological score and cerebral infarct volume (P<0.05) and improve the neuronal damage in ischemic cortex and hippocampus (P<0.05). The expression of NOX4, activated caspase-3(9), and Beclin 1 (P<0.05), as well as the LC3BII/I ratio, had been markedly elevated (P<0.05), while TSG administration could effectively suppress the expression of the above-mentioned proteins (P<0.05). In conclusion, TSG shows obvious protection against brain injury in I/R mice, and its mechanism may be related to suppressing the NADPH-induced OS and reducing neuronal apoptosis as well as autophagy.

## 1. Introduction

Cerebrovascular disease is the frequently occurring and common disease in clinic, which is characterized by the features of high disability rate and high mortality rate; among them, ischemic cerebrovascular disease has accounted for about 80% [1]. In clinic, the blood flow supply should be restored in the ischemic region of patient as soon as possible, so as to avoid further irreversible injury of cerebral tissue ischemia. However, it may also induce the more severe phenomenon, namely, the cerebral dysfunction, which is referred to as the ischemia/reperfusion (I/R) injury. The pathogenesis of I/R injury is extremely complicated, which has involved multiple pathological links, including energy metabolic disorder, local acidosis, inflammatory response, free radical injury, and induction of cell apoptosis and autophagy [2]. These pathological links do not exist independently; instead, they are intertwined and will induce a series of cascade reactions. At present, the effective drugs for treating brain I/R injury are lacking in clinic; therefore, research focus remains to search for the efficient and low-toxic new drugs for treating brain I/R injury in the current cerebral neurological disease field. Recent research suggests that numerous active ingredients in herbal medicine can attain satisfactory effect on treating brain I/R injury, which can provide a definite direction for the development of new drugs [3, 4].

Tetrahydroxystilbene glucoside (TSG) is the main active ingredient in the traditional herbal medicine* Fallopia multiflora*; previous studies have verified that it possesses extensive pharmacological activities, including antioxidation [5], anti-inflammation [6], reducing blood fat [7], and neuroprotection [8]. Interestingly, administration of TSG in APP695V717I transgene mice (transgene animal model of Alzheimer's disease, AD) can suppress the expression of Beclin 1 and LC3-II in hippocampal tissues of mice, which can thereby improve the learning, memory, and spatial orientation behavior of the APP695V717I transgene mice, suggesting that TSG can inhibit autophagy to exert the role of anti-AD [9]. However, no existing literature has reported the potential protection of TSG against brain ischemia/reperfusion (I/R) injury as well as the related neuroprotection mechanism.

In this paper, the middle cerebral artery occlusion (MCAO) model was utilized to prepare the I/R mice, so as to comprehensively evaluate the protection of TSG against brain injury in I/R mice. Moreover, the oxygen free radicals, SOD, and MDA levels were detected; and the expression of NOX4, Caspase-3, Caspase-9, Beclin 1, and LC3B was examined through Western blotting, so as to illustrate the potential protection mechanism of TSG against brain injury in I/R mice.

## 2. Materials and Methods

### 2.1. Animals

All animal procedures were conducted in accordance with the Guidelines for Care and Use of Laboratory Animals and were approved by the Animal Care and Use Committee at Anhui Medical University. KM adult male mice (25±2 g weight) were obtained from the Experimental Animal Center of the Anhui Medical University (SCXK (wan) 2013-002). Mice were housed in a standard environment (22±3°C with 55±10% controlled humidity and a 12-h dark/light cycle) with free access to food and water.

### 2.2. Construction of the Brain I/R Injury Mouse Model

The mouse brain I/R injury was achieved by the MCAO as described previously [10]. Briefly, KM mice were anesthetized by inhaled isoflurane and then fixed at the supine position and the body temperature was maintained at 37 ± 0.4°C with a heating blanket and thermocontrolled operating table. A blunt dissection was performed under a stereomicroscope, and the left common carotid artery (CCA), left external carotid artery (ECA), and left internal carotid artery (ICA) were exposed. CCA was temporarily occluded by a suture. ECA was permanently sutured as distally as possible. ICA was sutured distal to the bifurcation, followed by a small incision in the CCA between permanent and temporary sutures. The 6-0 surgical nylon filament was then advanced from the lumen of the external carotid artery into the internal carotid artery until resistance was felt, which ensured the occlusion of the origin of the middle cerebral artery (MCA) for 2 h, after which it was gently removed to allow reperfusion for 24 h. Sham group was subjected to the same surgical operation, but no suture occlusion was inserted.

### 2.3. Experimental Grouping and Administration

The healthy adult male KM mice were divided into 5 groups (n=25) according to the complete randomized group design method: sham group, model group (I/R), low dose TSG (catalog number 20170502; Dasf-Bio, Nanjing, China) group (3.0 mg/kg), moderate dose TSG group (6.0 mg/kg), and high dose TSG group (12.0 mg/kg) (Table 1). Time from the beginning of MCAO was recorded; 2 h after ischemia, the suture occlusion was removed to carry out I/R; and administration through tail vein injection was performed immediately at the time of reperfusion in mice. For the 3 TSG dose groups, corresponding doses were given, respectively, according to the above-mentioned drug doses, while equivalent volume of normal saline was given in sham group and model group.

### 2.4. Neurological Score

Upon the completion of reperfusion in mice, neurological score was assessed according to the Longa five-score method [11]. Mice with the neurological score of ≥1 point, with no basilar blood clot when taking out the brain and no arterial ring thrombosis were deemed to be successful modeling. The single blind method was adopted during the entire scoring process; in other words, the observers were not informed of the precise grouping of experimental animals.

### 2.5. Cerebral Infarct Volume Determined by TTC Staining

Cerebral infarct volume was determined 24 h after the MCAO with 2,3,5-triphenyltetrazolium chloride monohydrate (TTC; Sigma-Aldrich, USA) staining as described previously [10]. Briefly, mice were deeply anesthetized with isoflurane and then sacrificed by decapitation. The coronal brain slices (2-mm thickness) were cut, incubated in 1% solution of TTC for 15 min, and then fixed in 10% formaldehyde for 12 h. Images of each slice were captured using a digital scanner at 600 dpi. The percentage of infarct area was calculated by the Image J software.

### 2.6. Histopathological Examination

Mice were deeply anaesthetized with inhaled isoflurane 24h after MCAO and perfused with saline through the left cardiac ventricle, followed by fixing for 24h in 4% polyformaldehyde. Tissues were embedded in paraffin, and serial coronal sections of 5 *μ*m in thickness were prepared. After fixing for 24 h, the sections were processed by deparaffinization with xylene, rehydratation through graded alcohols, staining with hematoxylin and eosin (HE), and observation under a light microscope (BX-51; Olympus, Tokyo, Japan).

### 2.7. Detection of Free Oxygen Radicals in Brain Tissues

**Figure 1:**
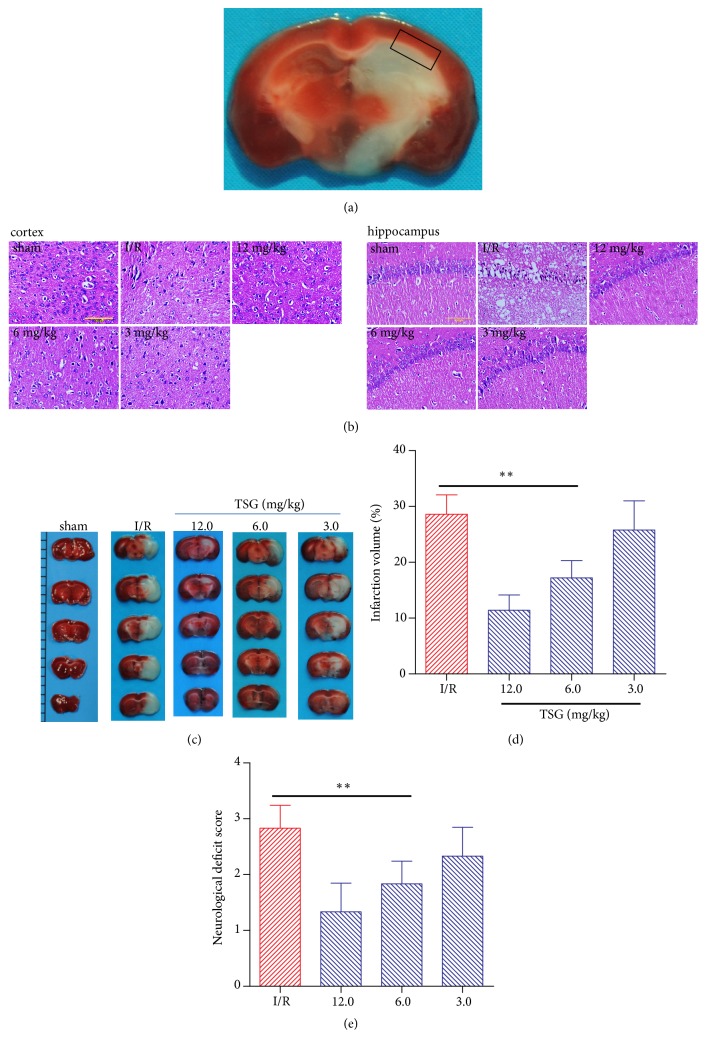
TSG injection reduced cerebral infarct volume proportion and improved neurological score. The brain tissues of ischemic penumbra were collected at 24h after MCAO. (a) The black frame indicated samples dissection and collection at the ischemic penumbra in the cortex and hippocampus around the ischemic boundary in this study. (b) Representative HE staining images of brain cortex and hippocampus in each group showed the pathological changes in ischemic penumbra (n=4). (c) Representative TCC staining graph of brain tissues in each group. (d) Cerebral infarct volume proportion in each group (n=6). (e) Longa neurological score in each group. Data are mean± SEM (n=6). ∗∗p<0.01 compared with I/R group.

24h after MCAO, the mice were sacrificed, the brains (removing the cerebrum and brainstem) were collected, and the ischemic penumbra in the cortex and hippocampus around the ischemic boundary (Figure 1(a)) was put into liquid nitrogen for measurement. The temperature in the resonant cavity of the JES-FA20000 spectrometer (JEOL) model ESR wavelength dispersive spectrometer (WDS) (Japanese Electronics) was adjusted to 130 K, the brain tissue specimens were taken out from the liquid nitrogen and rapidly put into the ESR resonant cavity, the temperature was balanced and maintained at 130 K, the ESR spectra were tested, and the specific software was utilized to analyze the major free radical signals in the spectra. Tissue oxygen free radical signal strength=total signal value/mass of the tested tissue.

### 2.8. Detection of SOD and MDA Levels in Brain Tissues

The brain tissues were obtained at 24h after MCAO. The supernatant of homogenate was prepared by centrifugation at 10000g × 10 min at 4°C. The SOD and MDA levels in the ischemic brain tissues were detected using commercial kits, respectively, according to the guidelines provided by the manufacturer. The SOD activity assay kit was purchased from Nanjing Jiancheng Bioengineering Institute (Cat. No. A001, Nanjing, China). This kit employed a xanthine–xanthine oxidase system to determine the inhibition of nitroblue tetrazolium (NBT) reduction due to generation of superoxide anions. The activity of SOD was expressed as units of nitrite per mg of protein. Total proteins were quantified by the classical Bradford method (Cat. No. P0006, Beyotime, Haimen, China). For the MDA detection, the assay kit was from Nanjing Jiancheng Bioengineering Institute (Cat. No. A003, Nanjing, China). The concentration of MDA was determined by the thiobarbituric acid method. It was assayed in the form of thiobarbituric acid reacting substances (TBARSs). The content of TBARS (used as an index of MDA) was expressed as nmol per mg protein.

### 2.9. Protein Expression Levels Detected through Western Blotting

Changes in the expression of NOX4 protein as well as Caspase-3, Caspase-9, Beclin 1, and LC3II/I in ischemic brain cortex and hippocampal tissues were detected using Western blotting, respectively. Then, the Bioshine ChemiQ 4600 fluorescence and chemiluminescence imaging system were adopted for developing, and the gray value of each band was calculated using the Image J analytical software.

### 2.10. Statistical Processing

SPSS 15.0 statistical software was adopted to analyze the experimental data, and all data were expressed as mean±SEM. Firstly, multiple groups of data were compared through one way analysis of variance (ANOVA); subsequently, the Tukey's method was utilized for pairwise comparison between two groups. The neurological score was evaluated using the Kruskal-Wallis analytical method, and pairwise comparison between groups was carried out using Mann-Whitey U test. A difference of P<0.05 was deemed statistically significant.

## 3. Results

### 3.1. TSG Reduced Cerebral Infarct Volume and Neurological Score

HE staining results suggested that (Figure 1(b)) the brain cortex neurons and neuroglia in sham group were normal, which were orderly arranged, the morphology of neurons was normal, and the capillary and small vessels had narrow lumen. In the I/R group, cells displayed various degrees of denaturation and necrosis, along with inflammatory neutrophil infiltration; interstitial cell (network-like) swelling and loosening; widened space around nerve cells, capillaries and small vessels; and glial cell swelling and enlarged cytoplasm transparent region. TSG could alleviate the above-mentioned pathological injuries in neurons in cortex and hippocampal CA1 region of the penumbra region to various degrees, improve neuron morphology, and alleviate nuclear pyknosis, inflammatory infiltration, and interstitial cell swelling. Brain tissue TTC staining results suggested that (Figures 1(c) and 1(d)) the brain sections in sham group were stained into the uniform dark red, with no infarct area observed; the cerebral infarct volume had accounted for a high proportion in the ischemic penumbra of mice in I/R model group, which had reached 28.63±3.43%; and TSG treatment could alleviate the cerebral infarct volume proportion in the ischemic penumbra of I/R mice in a dose-dependent manner (P<0.01). Moreover, Longa score results suggested that the neurological score in I/R model group was evidently increased, which had manifested obvious neurological injury; TSG treatment could reduce the neurological score and improve the neurological function in I/R mice (P<0.01, Figure 1(e)).

### 3.2. TSG Decreased the Oxygen Free Radical, MDA, and NOX4 Production, but Increased SOD Level in Ischemic Penumbra of I/R Mice

**Figure 2:**
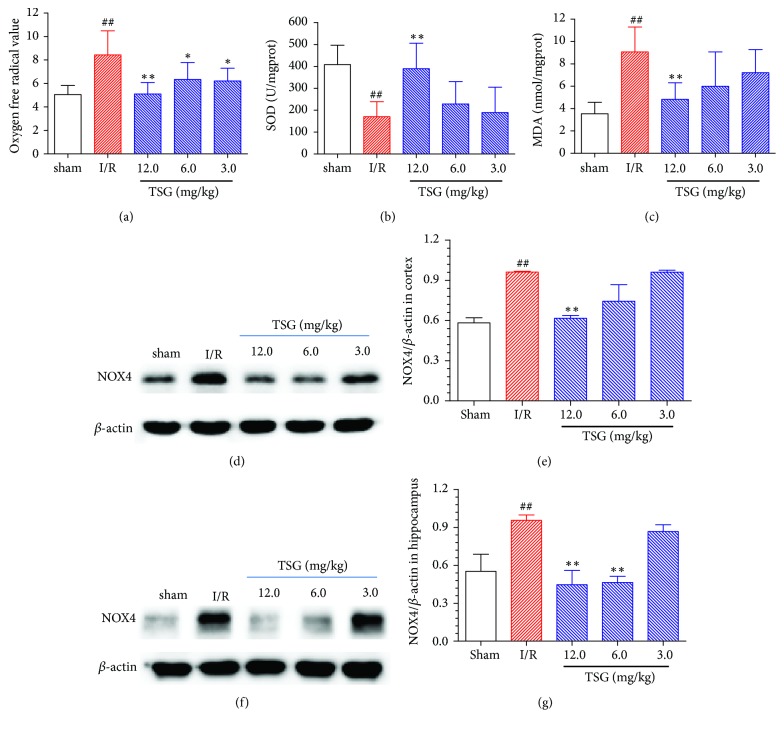
TSG decreased the oxygen free radical, MDA, and NOX4 production, but increased SOD level in ischemic penumbra of I/R mice. (a) Oxygen free radical levels in brain tissues of each group; (b) SOD levels in the brain tissues of each group; (c) MDA levels in brain tissues of each group. (d) Typical NOX4 protein expression graph in brain tissue cortex in each group. (e) Semiquantitative analysis results of NOX4 protein expression in brain tissue cortex. (f) Typical NOX4 protein expression graph in brain tissue hippocampus in each group. (g) Semiquantitative analysis results of NOX4 protein expression in brain tissue hippocampus. Data are mean±SEM (n=5).^ ##^p<0.01 compared with sham group. ∗∗p<0.01 compared with I/R group.

As shown in Figure 2(a), compared with Sham group, the oxygen free radical in the brain tissues of ischemic penumbra of I/R mice was markedly increased (P<0.01), while TSG could evidently suppress the production of oxygen free radicals in the brain tissues of ischemic penumbra of I/R mice (P<0.05). As shown in Figures 2(b) and 2(c), compared with Sham group, the SOD levels in the brain tissues of ischemic penumbra of I/R mice were markedly reduced (P<0.01), while the MDA levels were dramatically increased (P<0.01); meanwhile, TSG could improve the SOD levels and reduce the MDA levels in a dose-dependent manner (P<0.05). In addition, the NOX4 expression in cortex and hippocampus of ischemic penumbra in I/R group was upregulated (P<0.01); TSG could suppress NOX4 expression in cortical tissues in a dose-dependent manner (P<0.01); meanwhile, TSG (6.0 and 12.0 mg/kg) could notably suppress NOX4 expression in hippocampal tissue (P<0.01, Figures 2(d)–2(g)).

### 3.3. TSG Reduced Caspase-3 and -9 Expression in Cortex and Hippocampal Tissues in Ischemic Penumbra of I/R Mice

**Figure 3:**
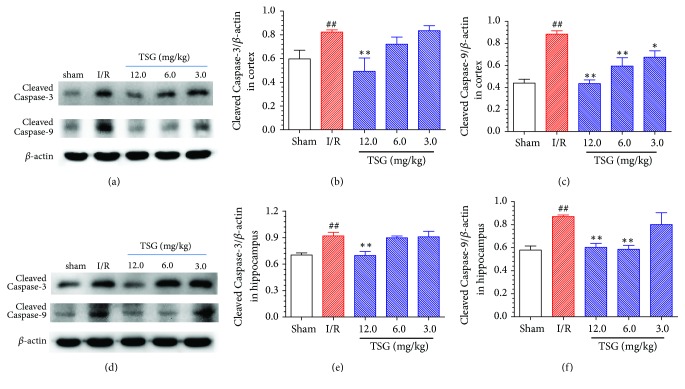
Effect of TSG on Caspase-3 and Caspase-9 protein expression in cortex and hippocampal tissues in ischemic penumbra of I/R mice. (a) Typical activated Caspase-3 and Caspase-9 protein expression in the brain tissue cortex of each group. (b) Semiquantitative analysis results of activated Caspase-3 protein expression in brain tissue cortex. (c) Semiquantitative analysis results of activated Caspase-9 protein expression in brain tissue cortex. (d) Typical activated Caspase-3 and Caspase-9 protein expression in the brain tissue hippocampus of each group. (e) Semiquantitative analysis results of activated Caspase-3 protein expression in brain tissue hippocampus. (f) Semiquantitative analysis results of activated Caspase-9 protein expression in brain tissue hippocampus. Data are mean±SEM (n=5). ^##^p<0.01 compared with sham group. ^∗∗^p<0.01 compared with I/R group.

It was shown in Figures 3(a)–3(c) that, compared with sham group, expression of the activated Caspase-3 and activated Caspase-9 in the cortical tissues of ischemic penumbra of I/R model group was evidently upregulated (P<0.01), TSG (12.0 mg/kg) could remarkably suppress the expression of activated Caspase-3 in cortical tissues of I/R mice, and TSG at each doses could notably inhibit Caspase-9 activation in the cortical tissues (P<0.05). As presented in Figures 3(d)–3(f), expression of the Caspase-3 and Caspase-9 activation in the hippocampal tissues of ischemic penumbra of I/R model group was dramatically upregulated (P<0.01), which could be markedly suppressed by the TSG treatment (P<0.01). The above results suggested that TSG could suppress the apoptosis of brain neurons in I/R mice, which could exert the protection of brain neurons in I/R mice.

### 3.4. Effect of TSG on the Expression of Beclin 1 and LC3B in Cortex and Hippocampal Tissues of Ischemic Penumbra in I/R Mice

**Figure 4:**
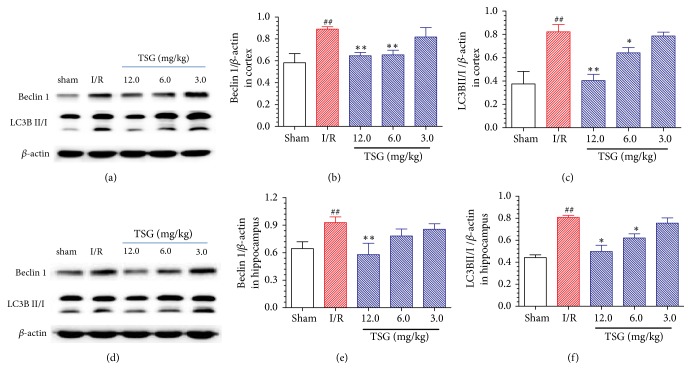
Effect of TSG on the expression of Beclin 1 and LC3B in cortex and hippocampal tissues of ischemic penumbra in I/R mice. (a) Typical Beclin 1 and LC3B protein expression in the brain tissue cortex of each group. (b) Semiquantitative analysis results of Beclin 1 protein expression in brain tissue cortex. (c) Semiquantitative analysis results of the LC3BII/I ratio in brain tissue cortex. (d) Typical Beclin 1 and LC3B protein expression in the brain tissue hippocampus of each group. (e) Semiquantitative analysis results of Beclin 1 protein expression in brain tissue hippocampus. (f) Semiquantitative analysis results of the LC3BII/I ratio in brain tissue hippocampus. Data are mean±SEM (n=5). ^##^p<0.01 compared with sham group. ^∗∗^p<0.01 compared with I/R group.

As shown in Figures 4(a)–4(c), compared with sham group, the Beclin 1 expression in cortical tissue in ischemic penumbra of I/R model group was upregulated (P<0.01), and the LC3BII/I ratio was increased (P<0.01); TSG could outstandingly suppress Beclin 1 expression in the cortical tissues of I/R mice and notably downregulate the LC3BII/I ratio (P<0.05). The Beclin 1 expression in hippocampal tissue in ischemic penumbra of I/R model group was also upregulated (P<0.01), and the LC3BII/I ratio was also increased (P<0.01), which could be both inhibited by the TSG treatment (P<0.05, Figures 4(d)–4(f)). The above results indicated that TSG could alleviate the neuron autophagy in I/R mice, thus exerting the neuronal protection of I/R mice.

## 4. Discussion

The morbidity of ischemic cerebrovascular disease is increasing year by year, which has become one of the major causes leading to neural dysfunction and death, thus bringing heavy burdens on both the patients and the medical health system. Previous studies have verified that ischemic cerebrovascular disease will result in the cascade reactions of vascular injury, blood brain barrier (BBB) destruction, and neuron injury due to blood flow restoration in the brain tissues [12]. Increasing efforts have been made to develop a new therapeutic strategy, but there is still a lot work to be done to reduce brain I/R injury, and there is no effective method to treat brain I/R injury. Previous studies have shown the neuroprotective effect of TSG by intraperitoneally and gavage administration on the MCAO rats [13–15]. This study had utilized the MCAO mice to explore the protection effect of TSG on the I/R injuries and the underlying mechanism. It was found that TSG administration via tail vein injection was more effective treatment with lower concentrations, compared with intraperitoneally and intragastric administration, and the treatment dose was optimized at 12.0 mg/kg. TSG treatment could reduce the cerebral infarct volume and Longa neurological score in I/R mice and alleviate the pathological injury in cortex and hippocampal CA1 region in ischemic penumbra in a dose-dependent manner, which had revealed the obvious protection effect of TSG on brain I/R injury in mice.

The pathophysiological mechanisms of brain I/R injury are extremely complicated, among which, oxidative stress response is considered to play a core role in the brain I/R injury [16]. Nox4/ROS plays key regulatory roles during oxidative stress process [17], for example, Nox4 belongs to the NADPH oxidase family member, which plays a major role in the pathological process of ROS production and neurodegeneration in ischemic stroke [18]. In the case of local brain ischemia, NOX4 expression in brain cortex was evidently elevated, leading to increased ROS production [19, 20]. Excessive ROS will be produced during the I/R process, which will excessively consume the endogenous antioxidase in the body, reduce the activity of antioxidase, and decrease the ROS scavenging capacity of brain tissue. Besides, the excessively accumulated ROS may lead to fat oxidation, protein oxidation and DNA damage, and energy metabolic failure, finally leading to brain neuron death and apoptosis. SOD is the most important defending enzyme in the body to scavenge oxygen free radicals and block the free radical pathological chain reaction; typically, the SOD activity can serve as a major index to measure the oxygen free radical scavenging capacity [21]. A large number of lipid peroxides will be formed in the chained lipid peroxidation reaction induced when the cells are attacked by free radicals, among which, MDA has the greatest toxicity, and the changes in its content can indirectly reflect the changes in oxygen free radical content and the degree of injury to tissues [22]. In this study, NOX4 expression in the cortex and hippocampal tissues of ischemic penumbra of I/R mice was markedly upregulated, production of the oxygen free radicals was evidently increased, and SOD level was dramatically reduced, while MDA level was markedly elevated; moreover, TSG administration could downregulate NOX4 expression, oxygen free radical production, and MDA level in brain tissues of I/R mice, while elevating the SOD level in brain tissues of I/R mice, which had suggested that TSG could regulate Nox4/ROS to exert the antioxidation, thus mitigating the brain I/R induced injury.

A series of research has indicated that oxidative stress will induce cell apoptosis, while neuronal apoptosis plays a crucial role in the genesis and development of ischemic brain injury [2, 23]. Reversing the ischemia-induced neuronal apoptosis can alleviate the neurological deficit in mice [24]. The aspartic acid-specific cysteine protein kinase family (Caspase) is the most important protease during the cell apoptosis process: Caspase-3 and Caspase-9 are the most crucial proteases in the cell apoptosis mitochondrial pathway, while Caspase-3 is the most critical apoptosis-executing protease in the downstream of Caspases cascade reaction [25]. Previous studies also verify that suppressing the caspase-3 activity can protect brain neurons under I/R status [26]. Results of this study suggested that the activated Caspase-3 and Caspase-9 expression in the penumbra cortex and hippocampal tissues in I/R model group was markedly upregulated, which had revealed that the cell apoptosis reaction in penumbra of I/R model group was increased, thus closely participating in the early brain tissue acute injury after reperfusion. TSG administration could effectively suppress the activated Caspase-3 and Caspase-9 expression in the cortex and hippocampal tissues of I/R mice, suggesting that TSG could suppress brain neuron apoptosis in I/R mice and exert protection of the brain neurons in I/R mice.

Autophagy is a programmed cell survival process, which can respond to various stresses, such as oxidative stress, nutrient deficiency, and ischemia, through the degradation of intracellular cytoplasm macromolecules and organelles by the lysosome system; therefore, autophagy plays a key role in maintaining cell metabolism and homeostasis [27]. Appropriate autophagy can protect nerve cells through providing free fatty acid, amino acid, and nucleotide and scavenging the damaged organelles; however, the long-term upregulation of autophagy will induce excessive degradation of cellular contents, thus triggering cell death and aggravating brain I/R injury [28]. An increasing number of studies have suggested that the excessive activation of autophagy may aggravate brain I/R injury [29]. LC3B (the most important subtype of LC3) is the marker molecular of autophagosome, which is one of the vital markers reflecting whether autophagy is activated. During the autophagy process, the LC3BI level is reduced, while LC3BII expression is rapidly elevated; therefore, determining the intracellular LC3BII expression quantity or LC3BII/I ratio has become the major criterion for judging the autophagy level [30]. Beclin 1 is one of the key proteins regulating autophagy, which can trigger the protein cascade involved in autophagosome formation to directly exert its function [31]. In this experiment, Beclin 1 expression, the active marker of autophagosome, was markedly elevated in the cortex and hippocampal tissues of I/R mice, and the LC3BII/I ratio was also dramatically elevated, while TSG administration could effectively reduce Beclin 1 expression and the LC3BII/I ratio in I/R mice, which suggested that TSG could alleviate brain neuronal autophagy in I/R mice, thus exerting the protection of brain neurons in I/R mice.

## 5. Conclusion

TSG, the main active ingredient of herbal medicine* Fallopia multiflora*, can effectively alleviate brain I/R injury and mitigate the neurological defect symptoms. A previous study has highlighted the neuroprotective effect of TSG on cerebral ischemia, which was regulated by inhibiting iNOS, JNK, and NF-*κ*B and activating SIRT1 [13]. Our current study found that the potential mechanism of TSG in brain I/R injuries may be to regulate the antioxidation of Nox4/ROS, reduce the neuronal apoptosis in I/R mice, and suppress the excessive neuronal autophagy in I/R mice. This study can provide certain foundation for the clinical application of TSG in treating ischemic cerebrovascular disease. However, the internal association and mechanism of TSG in regulating oxidative stress, autophagy, and apoptosis during the brain I/R injury process should be further illustrated.

## Figures and Tables

**Table 1:** Number of rats killed for each test in different groups.

	TTC staining	HE staining	Free radical test	SOD and MDA detection	Western blot	Total
Sham groups	6	4	5	5	5	25
I/R groups	6	4	5	5	5	25
TSG (12.0 mg/kg)	6	4	5	5	5	25
TSG (6.0 mg/kg)	6	4	5	5	5	25
TSG (3.0 mg/kg)	6	4	5	5	5	25

## Data Availability

The data used to support the findings of this study are available from the corresponding author upon request.
